# Case Report: Heparin resistance as the harbinger of heparin-induced thrombocytopenia in acute pulmonary embolism

**DOI:** 10.3389/fmed.2026.1818171

**Published:** 2026-06-18

**Authors:** Na Yu, Fengqiang Wang

**Affiliations:** Department of Pulmonary and Critical Care Medicine, Liaocheng People's Hospital, Liaocheng, Shandong, China

**Keywords:** acute pulmonary embolism, case report, direct thrombin inhibitors, heparin resistance, heparin-induced thrombocytopenia, unfractionated heparin

## Abstract

Heparin-induced thrombocytopenia (HIT) is a severe, prothrombotic complication of heparin therapy. While thrombocytopenia is a hallmark, diagnosis can be obscured when preceded by unexplained heparin resistance and progressive thrombosis. We present the case of a 50-year-old man with intermediate- to high-risk acute pulmonary embolism (PE), deep vein thrombosis and a right ventricular mass. Despite timely and progressive dose escalation of intravenous unfractionated heparin [UFH; up to 1,850 U/h (~24.7 U/kg/h)], monitoring by activated partial thromboplastin time (APTT) revealed a failure to achieve therapeutic anticoagulation (heparin resistance), and he suffered two recurrent episodes of hemodynamic instability on treatment days 3 and 6, requiring rescue thrombolysis. Follow-up imaging revealed thrombus progression. On day 8 of heparin therapy, thrombocytopenia (platelets 104 × 10^9^/L) and a high 4Ts score (6 points) prompted HIT suspicion. Heparin was immediately discontinued and replaced with intravenous argatroban (initiated at 2 μg/kg/min), resulting in prompt attainment of the target therapeutic APTT range. HIT was confirmed by positive anti-platelet factor 4 (PF4)/heparin immunoglobulin G (IgG) antibodies. After platelet count recovery (to >150 × 10^9^/L), argatroban was discontinued and anticoagulation was switched to oral rivaroxaban. Follow-up studies at discharge and 3 months showed significant thrombus resolution, with normalized cardiac function and complete resolution of the intracardiac mass. This case underscores that unexplained heparin resistance with thrombotic progression, even preceding significant thrombocytopenia, a recognized harbinger of HIT-associated thrombosis, is a critical indicator of HIT. Immediate diagnostic evaluation and switch to non-heparin anticoagulants are imperative to avert life-threatening complications.

## 1 Introduction

Heparin-induced thrombocytopenia (HIT) is a severe adverse drug reaction characterized by pathogenic platelet activation, leading to a profound prothrombotic state (1). The classic diagnostic paradigm hinges on a significant platelet count fall occurring 5–10 days after heparin exposure (1). However, clinical recognition is often delayed in atypical presentations, posing a substantial risk for life-threatening thrombotic complications (2, 3).

One such diagnostic challenge arises when unexplained heparin resistance—defined clinically in this case by the failure to achieve target anticoagulation despite escalating intravenous unfractionated heparin to a maximum dose of 24.7 U/kg/h (corresponding to 44,400 U per day), a total daily dose that exceeds the conventional threshold of 35,000 U often cited for heparin resistance—accompanies thrombotic events. While heparin resistance has multiple etiologies, its occurrence in the setting of new or progressive thrombosis should raise immediate suspicion for HIT, as anti-platelet factor 4 (PF4)/heparin antibodies can trigger platelet activation and the release of PF4, which avidly binds to and neutralizes circulating heparin, thereby attenuating its anticoagulant effect (4). Despite its critical implication, this early warning sign is frequently overlooked, leading to a delayed diagnosis and the continued administration of heparin, which exacerbates the thrombotic risk, rather than prompting a timely diagnostic evaluation for HIT.

Herein, we report an instructive case of acute pulmonary embolism in which persistent heparin resistance and recurrent thrombosis were the sentinel features, preceding the development of overt thrombocytopenia. This case underscores the imperative for clinicians to recognize this atypical sequence, as immediate suspicion and switch to a non-heparin anticoagulant are crucial to avert catastrophic outcomes.

## 2 Case presentation

A 50-year-old man presented to our emergency department with syncope preceded by 1 week of paroxysmal chest tightness. Initial evaluation at a local hospital revealed right heart enlargement, a hyperechoic right ventricular mass (suspected thrombus or myxoma), moderate pulmonary hypertension (Pulmonary Artery Systolic Pressure (PASP) 55 mmHg), and evidence of multiple chronic cerebral infarcts on cranial CT. His medical history was significant for hypertension and lower extremity varicose veins.

On admission, vital signs were as follows: blood pressure 138/100 mmHg; heart rate 119 beats/min; respiratory rate 16 breaths/min; pulse oximetry on room air, 96%; temperature 36.6 °C; the patient weighed 75 kg. Chest auscultation revealed no abnormalities, and cardiac, abdominal, and neurological examinations were unremarkable. Lower limb examination revealed significant right-sided swelling, with a 2-cm increase in thigh circumference and 0.5-cm increase in calf circumference compared to the left, along with prominent varicose veins.

Laboratory findings on admission revealed leukocytosis (10.85 × 10^9^/L) with neutrophilia (76.7%) and lymphocytopenia (16.20%), a normal platelet count (233 × 10^9^/L), and an elevated C-reactive protein (61.85 mg/L). A markedly elevated D-dimer (29,900 μg/L) was measured using the HemosIL^®^ D-Dimer HS 500 assay, a latex-enhanced immunoturbidimetric method, on an ACL TOP series coagulation analyzer; results reported in fibrinogen equivalent units [FEU], and an elevated N-terminal pro-B-type natriuretic peptide level (7,610 pg/ml) were also noted, with a normal troponin I. Serological, immunological, and thrombophilia screenings to exclude infection, malignancy, autoimmune, and hereditary thrombophilia disorders were unremarkable. Computed tomography pulmonary angiography (CTPA; Figure 1) showed multiple low-attenuation filling defects in the bilateral pulmonary arteries and their branches. Echocardiography demonstrated right ventricular dysfunction (tricuspid annular plane systolic excursion 14 mm), moderate pulmonary hypertension (PASP 55 mmHg), and a mobile intracardiac mass (13 × 15 mm). Compression (duplex) ultrasonography of the right lower extremity revealed deep vein thrombosis in the right popliteal vein, accompanied by thrombosis in the proximal segment of the great saphenous vein (superficial vein thrombosis).

**Figure 1:**
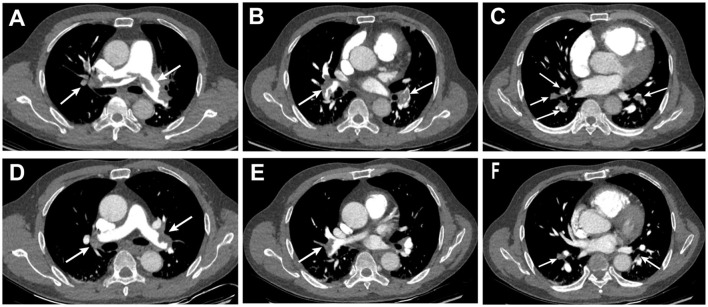
Serial computed tomography pulmonary angiography (CTPA) findings in acute pulmonary embolism. **(A–C)** Initial CTPA upon admission demonstrates extensive bilateral pulmonary embolism, with multiple filling defects (arrows) visible within the main, lobar, and segmental pulmonary arteries. **(D–F)** Follow-up CTPA was performed to assess treatment response (arrows).

The nature of the intracardiac mass (thrombus vs. myxoma) remained uncertain on echocardiography. We discussed this finding with the patient and his family. Given the extensive pulmonary embolism (including saddle-type involvement) and the patient's intermediate-high risk status, proceeding immediately with further contrast-enhanced imaging (such as cardiac CT or magnetic resonance) was considered to carry non-negligible procedure-related risks in this acute setting. These risks included potential contrast-induced nephropathy, the hazards of transporting an unstable patient, and the critical delay in initiating definitive antithrombotic therapy. After shared decision-making, the patient and his family agreed to a management plan of initiating immediate therapeutic anticoagulation coupled with close serial echocardiographic monitoring to assess the evolution of the cardiac mass.

The patient was admitted on the evening of January 23, 2023. Following initial management in the emergency department, which included a single subcutaneous dose of low-molecular-weight heparin (LMWH) 5,000 AXa units (Low-molecular-weight Heparin Calcium Injection; Shenzhen Seabor Biopharmaceutical Co., Ltd., China), therapeutic intravenous unfractionated heparin (UFH, Heparin Sodium Injection, 12,500 units/2 ml; Hebei Changshan Biochemical Pharmaceutical Co., Ltd., China) was commenced immediately on day 0 (January 23, 2023) at 1,000 U/h (approximately 13.3 U/kg/h) and titrated according to a weight-based nomogram (Table 8 from the Chinese guideline for the diagnosis and prevention of pulmonary thromboembolism, 2018) (5), which specifies both infusion rate adjustments and supplemental boluses based on activated partial thromboplastin time (APTT) monitoring. A detailed timeline of key clinical events, laboratory parameters, and interventions is provided in Supplementary Table S1.

Laboratory monitoring and treatment response. UFH therapy was monitored via serial measurements of the APTT. APTT monitoring for UFH was performed on blood samples collected in 3.2% sodium citrate tubes. Plasma was separated by centrifugation at 2,000 g for 10 min within 1 h of blood collection. Following centrifugation, the plasma was not transferred to a separate tube; instead, the original tube was directly loaded onto the automated coagulation analyzer (ACL TOP 700 series) for testing, which was performed immediately (always within 1 h of centrifugation) according to the laboratory's standard protocol. Analyses were conducted using the SynthASil reagent. The institutional therapeutic target for UFH was an APTT ratio of 1.5 to 2.5 times the median of the normal reference range. The laboratory's normal reference range for APTT (25.1–36.5 s) was established by the reagent manufacturer for the specific analyzer and was confirmed through in-house verification using a local cohort of healthy donors. The infusion rate was titrated according to the nomogram, with APTT assessments performed every 4–6 h during dose adjustments. Despite protocol-adherent dose escalation to a maximum of 1,850 U/h (~24.7 U/kg/h), the target APTT range was never consistently achieved (Figures 2, 3), meeting the clinical definition of heparin resistance. The patient subsequently experienced two distinct episodes of hemodynamic instability on day 3 (January 26) and day 6 (January 29) of UFH therapy. Both episodes, characterized by systemic hypotension (with blood pressures of 75/52 and 85/45 mmHg, respectively) and hypoxemia (PaO_2_/FiO_2_ 98 mmHg during the second episode), met the criteria for high-risk pulmonary embolism, necessitating rescue thrombolysis with recombinant tissue plasminogen activator (rt-PA, 50 mg). Follow-up studies confirmed thrombotic progression despite maximal anticoagulation. While echocardiography demonstrated worsened right ventricular function (TAPSE 9 mm) and an enlarged (77 × 10 mm) mobile cardiac mass, follow-up compression ultrasonography of the right lower extremity demonstrated thrombus progression, with proximal extension into the right femoral vein.

**Figure 2:**
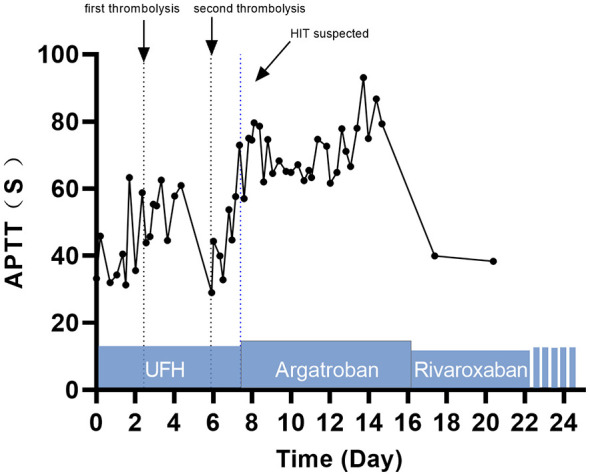
Dynamic changes in activated partial thromboplastin time (APTT) during anticoagulation therapy

**Figure 3:**
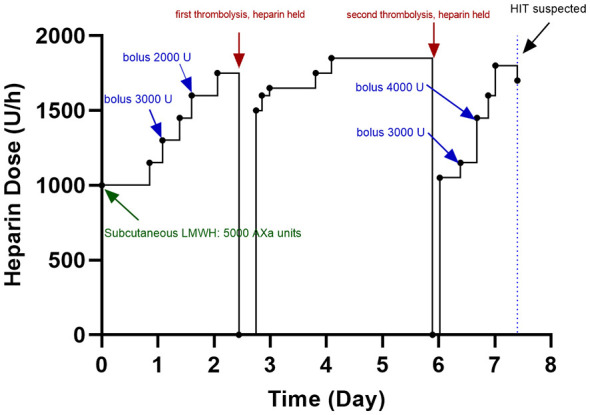
Timeline of anticoagulant therapy. The solid line represents the continuous intravenous unfractionated heparin (UFH) infusion rate (U/h), adjusted based on clinical monitoring. A green arrow indicates the initial subcutaneous loading dose of low-molecular-weight heparin (LMWH) in the emergency department (5,000 AXa units). Dark blue arrows and labels indicate additional bolus doses of UFH administered when activated partial thromboplastin time (APTT) targets were not met. Red arrows mark the initiation of thrombolysis (rt-PA) and the corresponding periods when the UFH infusion was temporarily held. A black arrow indicates the time point when heparin-induced thrombocytopenia (HIT) was suspected clinically.

Serial platelet monitoring (initially sparse, then intensified to daily or twice-daily upon clinical suspicion) revealed a concerning trend. The baseline platelet count was 233 × 10^9^/L (day 0). A progressive decline was observed, with the platelet count falling to 104 × 10^9^/L on day 8 of UFH therapy, reaching a nadir of 81 × 10^9^/L on day 9 (Figure 4; see Supplementary Table S2 for detailed timepoints). The finding of thrombocytopenia (platelets 104 × 10^9^/L) on day 8 (January 31), in conjunction with a high-probability 4Ts score of 6 points (calculated as follows: Thrombocytopenia, 2 points [fall >50% from baseline (233 to 104 × 10^9^/L) with nadir ≥20 × 10^9^/L]; Timing, 2 points (onset on day 8, within 5–10 days of heparin initiation); Thrombosis, 1 point [for progressive thrombosis, evidenced by extension of deep vein thrombosis on venous duplex ultrasound and worsened right ventricular function with enlargement of a mobile intracardiac mass on echocardiography, indicating systemic thrombotic progression]; Other causes, 1 points [while the clinical picture was most consistent with HIT, the patient received concomitant medications (e.g., propranolol, torasemide) with documented, albeit rare, association with thrombocytopenia, precluding a definitive 2-point assignment)] raised strong suspicion for HIT. Antibodies against PF4/heparin complexes [immunoglobulin G (IgG) class] were sent for analysis.

**Figure 4:**
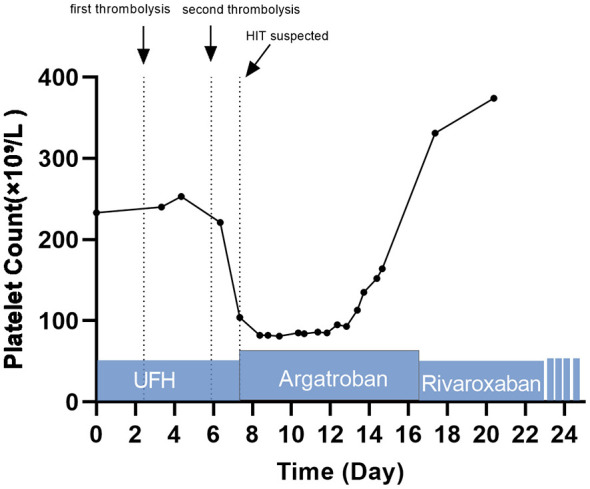
Dynamic changes in platelet count during anticoagulation therapy.

Upon strong suspicion of HIT, intravenous UFH was immediately discontinued and replaced with the direct thrombin inhibitor argatroban. Argatroban was administered as a continuous intravenous infusion starting at 2 μg/kg/min, with the dose titrated to maintain the APTT within a target range of 1.5 to 3.0 times the patient's baseline, using the SynthASil reagent on an ACL TOP 700 LAS analyzer. Therapeutic APTT was promptly achieved. The diagnosis was subsequently confirmed by a positive anti-PF4/heparin IgG antibody test performed at JINAN KINGMED CENTER FOR CLINICAL LABORATORY, using a commercial enzyme-linked immunosorbent assay (ELISA) kit manufactured by Beijing Bofurui Gene Diagnostics Technology Co., Ltd., with a quantitative optical density (OD) value at 492 nm of 1.92 (reference range < 0.40), establishing acute HIT type II complicating high-risk pulmonary embolism. After platelet count recovery to above 150 × 10^9^/L, argatroban was discontinued and anticoagulation was switched to oral rivaroxaban (15 mg twice daily). Prior to discharge, follow-up CTPA demonstrated improvement in the bilateral pulmonary emboli (Figures 1D–F). Lower extremity venous ultrasonography revealed partial recanalisation of the right deep vein thrombosis. Echocardiography showed normal cardiac chamber dimensions, coordinated wall motion without regional abnormalities. No intracardiac masses were visualized. Three months post-discharge, repeat CTPA showed a residual low-attenuation filling defect in the right upper lobe pulmonary artery branch, with complete resolution of thrombus in the pulmonary trunk and other major branches.

## 3 Discussion

HIT is a severe, IgG-mediated adverse drug reaction characterized by pathological platelet activation. Its incidence ranges from 1%−5% in patients receiving UFH, declining to 0.1–1% with low-molecular-weight heparin (2). HIT arises when antibodies recognize complexes of PF4 and heparin, triggering robust platelet activation and a prothrombotic state that culminates in venous or arterial thrombosis. The clinical consequences are grave, with untreated HIT associated with a 50% risk of thromboembolic complications—including pulmonary embolism, limb ischemia, myocardial infarction, and stroke—and a mortality rate of 5–10% (1).

The diagnosis of HIT requires integrated clinical assessment and laboratory confirmation. Clinically, the 4Ts scoring system is first applied to evaluate the pretest probability (6). The 4Ts score should be applied dynamically upon any unexplained clinical deterioration (e.g., new/progressive thrombosis or heparin resistance) during heparin therapy, rather than statically only after overt thrombocytopenia is confirmed. In this case, a formal score of 6 (high probability) was calculated on day 8, prompted by the confirmed platelet decline to 104 × 10^9^/L. However, a high index of suspicion was warranted earlier. On day 6, following the second episode of hemodynamic instability with imaging-confirmed thrombotic progression despite escalated UFH, the platelet count (221 × 10^9^/L) was still within the normal range. Applying the 4Ts principles at that earlier juncture (day 6, platelet count 221 × 10^9^/L) would have yielded an intermediate-to-high probability score: Thrombocytopenia, 0 points (platelet count normal); Timing, 2 points (onset of progressive thrombosis on day 6, within 5–10 days of heparin initiation); Thrombosis, 1 point (for progressive thrombosis, evidenced by recurrent hemodynamic compromise and imaging-confirmed thrombus extension); Other causes, 1 point (concomitant medications with rare association to thrombocytopenia were present). This hypothetical score of 4 points underscores the importance of early application to facilitate diagnostic evaluation.

Laboratory testing comprises two modalities: (1) antigen assays (e.g., PF4/heparin enzyme-linked immunosorbent assay [ELISA]), which exhibit high sensitivity but moderate specificity, with IgG-specific ELISA offering superior specificity (7); and (2) functional assays (e.g., serotonin release assay [SRA]), considered the diagnostic gold standard due to high sensitivity and specificity (8), though technically demanding. The assay in our case was performed by an external laboratory (JINAN KINGMED center for clinical laboratory) and provided a quantitative OD value at 492 nm. Research indicates that higher OD values are associated with an increased risk of clinical HIT and thrombotic events (9).

While thrombocytopenia is the hallmark presentation, thrombosis precedes platelet reduction in a significant proportion of cases. A detailed cohort analysis by Greinacher et al. (3) found that among 209 HIT patients with thrombosis, the thrombotic event occurred before a >50% platelet count decline in 33.5% of cases (70 patients), and on the same day in an additional 26.3%. This indicates that in nearly 60% of HIT-associated thromboses, the classic sequence is altered, reinforcing that clinical suspicion should be triggered irrespective of the absolute platelet count. Consistent with this classic observation, in our patient, progressive thrombosis and heparin resistance were evident on day 6, while the platelet count (221 × 10^9^/L) was still within the normal range (i.e., >150 × 10^9^/L) and had not yet shown a >50% decline from baseline.

Heparin resistance is defined as the failure to achieve target anticoagulation levels despite administration of appropriate doses of UFH. While traditional daily dosing criteria (>35,000 U/d) remain widely used, weight-based thresholds (>30 U/kg/h) are increasingly recommended, though a consensus definition is lacking (10). An International Society on Thrombosis and Haemostasis survey reveals divergent monitoring practices, with approximately 50% of centers relying on APTT and 50% utilizing anti-factor Xa (anti-Xa) activity assays (11). Given its superior specificity for quantifying heparin concentration, anti-Xa activity monitoring is the preferred method to confirm true laboratory heparin resistance, especially when APTT results appear discordant with the clinical picture (12). This is particularly relevant because APTT, while widely available, can be influenced by elevated factor VIII, lupus anticoagulant, or other acute-phase reactants, potentially leading to a falsely low value that does not accurately reflect the heparin effect. In our patient, the diagnosis of heparin resistance was initially based on the persistent failure to achieve the target APTT despite dose escalation.

The temporal relationship and nature of heparin exposure in this case strongly implicates ongoing intravenous unfractionated heparin (UFH) as the causative agent for HIT. The patient received a single dose of LMWH in the emergency department prior to admission. While LMWH can also induce HIT, its risk is substantially lower than that of UFH. The characteristic platelet count decline commenced on day 8 of continuous, high-dose intravenous UFH therapy, which aligns precisely with the typical 5–10 day window for “typical-onset” HIT. Furthermore, the profound and progressive “heparin resistance” observed is a hallmark more commonly associated with UFH-triggered HIT due to the stronger binding affinity of UFH for PF4. Therefore, the clinical and laboratory timeline is most consistent with HIT precipitated by the sustained intravenous UFH infusion, with the prior LMWH exposure unlikely to be the primary trigger. Therefore, the observed heparin resistance, accompanied by progressive thrombosis, supports the interpretation that it served as a critical early diagnostic clue for HIT in this context.

It is important to critically examine the term “heparin resistance” as used in this context. Recent authoritative commentaries have highlighted that the concept is heavily challenged (13–15). Strictly speaking, pharmacological “resistance” implies a failure of the drug to act on its target, which is not typically the case with heparin, as increasing doses can often overcome a subtherapeutic response. Therefore, the phenomenon is more accurately described as a “diminished laboratory response” or “clinical failure to achieve target anticoagulation.” Furthermore, there is no consensus on its definition, and this response is not specific to HIT, occurring in other hypercoagulable states (e.g., malignancy, extensive thrombosis) mediated by mechanisms like PF4 release or acute-phase reactions (10).

In our patient, this “clinical failure” manifested as the inability to achieve target APTT despite escalating UFH to 24.7 U/kg/h (44,400 U/day), a dose that met the conventional daily dose criterion for resistance. We use the term pragmatically to describe this objective observation. Critically, when this laboratory and clinical evidence of heparin resistance is accompanied by progressive thrombosis—as it was in our case on days 3 and 6—it should be interpreted not as a diagnosis, but as a cardinal warning sign mandating immediate investigation, with HIT high in the differential.

This presentation is consisten with the classic description of HIT. The finding historically termed “heparin resistance” is a recognized hallmark of HIT-associated thrombosis, reflecting the failure to achieve target anticoagulation despite increasing heparin doses, a phenomenon well-documented in early case series (16, 17). Mechanistically, in HIT, this resistance is understood to stem primarily from the immune-mediated release of PF4, which avidly binds and neutralizes circulating heparin (18–20). This evidence of resistance often precedes or coincides with the onset of new thrombosis and can manifest before overt thrombocytopenia develops, serving as a critical early diagnostic clue (16, 18). In our patient, this temporal sequence was strikingly evident and heralded the subsequent platelet count decline, underscoring the predictive value of this clinical observation.

When clinical suspicion for HIT is high—typically indicated by an intermediate- or high-probability 4Ts score—management must promptly shift from diagnostic suspicion to preemptive intervention. The cornerstone of this approach is the immediate discontinuation of all heparin-based anticoagulants coupled with the simultaneous initiation of a non-heparin alternative. This strategy aims to rapidly mitigate the pathological thrombin burst mediated by HIT antibodies while awaiting laboratory confirmation, thereby preventing progression to life-threatening thrombotic events.

In this high-risk setting, direct thrombin inhibitors (DTIs), such as argatroban or bivalirudin, serve as the preferred initial therapy due to their targeted mechanism of action (10). These agents directly and reversibly inhibit thrombin (factor IIa) activity, producing a potent anticoagulant effect independent of AT, which is often consumed in HIT. In this case, the anticoagulant effect of argatroban was managed by monitoring the APTT. Although assays such as the dilute thrombin time (dTT) or ecarin clotting time (ECT) are considered more specific for monitoring direct thrombin inhibitors (21), they are not routinely available in most clinical laboratories (21). Consequently, APTT remains the most practical and widely adopted test for real-time dose titration in acute settings, despite its limitations of non-linear dose-response and reagent-dependent variability, which necessitates institution-specific validation of the therapeutic range (21).

Our monitoring practice adhered to the Chinese Guidelines for the Diagnosis, Treatment and Prevention of Pulmonary Thromboembolism (5), targeting an APTT of 1.5 to 3.0 times the patient's baseline value. The patient's pre-anticoagulation baseline APTT was 33.2 s, thus the individualized target range was set at 49.8 to 99.6 s. APTT was measured using the clot-based method with SynthASil reagent on an ACL TOP 700 LAS analyzer (normal lab range: 25.1–36.5 s). As shown in Figure 2, the dynamic APTT profile demonstrated a rapid and stable achievement of this target during argatroban titration. This stands in stark contrast to the “heparin resistance” observed with UFH and confirms the effectiveness of APTT-based monitoring in guiding therapy for this complex case of HIT.

Agent selection requires individualization: bivalirudin, cleared renally, may be preferred in hepatic impairment, whereas argatroban, metabolized hepatically, is often chosen in renal dysfunction (22). This pathophysiology-driven, preemptive strategy establishes a critical “anticoagulation safety window.” In the context of HIT, especially when heralded by heparin resistance, the pathologic thrombin burst is already active, driving both thrombosis and heparin consumption. Any delay in establishing effective alternative anticoagulation (e.g., by persisting with heparin dose escalation) directly extends this window of uncontrolled thrombin generation, dramatically increasing the risk of new or progressive, potentially fatal, thrombosis. The immediate switch to a non-heparin anticoagulant is therefore paramount to rapidly close this window. After platelet count recovery (typically >150 × 10^9^/L) and diagnostic confirmation, transition to a direct oral anticoagulant (DOAC) for extended therapy is considered (22). If a vitamin K antagonist (e.g., warfarin) is selected, its initiation must be overlapped with an effective non-heparin parenteral agent only after platelet recovery to avoid an early prothrombotic risk (23). The prognosis of HIT is highly dependent on the timeliness of diagnosis and intervention. Early recognition and appropriate management significantly reduce both thrombotic risk and mortality (1). In cases like ours, where heparin resistance precedes thrombocytopenia, a high index of suspicion upon this early signal is the critical determinant of a favorable outcome. The immediate discontinuation of heparin and switch to a non-heparin agent, as performed, effectively interrupts the pathological cascade before it culminates in catastrophic thrombosis.

This report has several limitations that warrant discussion. First, a confirmatory functional platelet activation assay (e.g., SRA) was not performed. While the combination of a high-probability 4Ts score (6 points) and a positive IgG-specific anti-PF4/heparin immunoassay (with a high OD value) provides strong supportive evidence for HIT according to current diagnostic algorithms, SRA remains the diagnostic gold standard for specificity. Its absence introduces a marginal degree of diagnostic uncertainty, albeit within the constraints of real-world clinical practice where such specialized testing is often unavailable. Our diagnostic approach, which integrated high-quality clinical data with the best available serological test, aligns with guideline-endorsed management strategies when functional testing is inaccessible. Second, the timing of the first thrombotic event (day 3 of therapy) in a patient without documented recent heparin exposure is noteworthy. Classic “typical-onset” HIT manifests 5–10 days after initiation. The early occurrence could raise consideration for “rapid-onset” HIT (which requires recent prior exposure) or other prothrombotic states. However, the subsequent clinical trajectory—characterized by progressive heparin resistance, a second thrombotic event, and a delayed but significant platelet count decline beginning on day 8—forms a sequence highly characteristic of an evolving HIT immune response. This pattern underscores that the prothrombotic state and its laboratory correlate (heparin resistance) can precede overt thrombocytopenia, a key teaching point of this case. Third, regarding the attribution of HIT etiology, the patient received both a single dose of LMWH in the emergency department and subsequent continuous intravenous UFH. Although the temporal evidence (platelet decline on day 8 of UFH therapy) strongly implicates ongoing UFH as the primary trigger, a contributory role of the prior LMWH exposure cannot be entirely excluded on pathophysiological grounds. Fourth, and pertinent to the central teaching point, heparin resistance was defined and monitored using the APTT rather than the more specific anti-Xa activity assay. As discussed, APTT can be influenced by various factors unrelated to heparin concentration, such as elevated factor VIII and other acute-phase reactants which are common in critically ill patients. Concurrent anti-Xa monitoring would have provided a more direct quantification of heparin levels. In the setting of HIT, where PF4-mediated heparin neutralization is the suspected mechanism, both APTT and anti-Xa levels would be expected to be low. However, the anti-Xa assay would have more specifically confirmed that the subtherapeutic APTT was due to true heparin depletion (e.g., by PF4 neutralization) rather than an artifact of APTT interference. The reliance on APTT alone reflects real-world clinical constraints but is a recognized methodological limitation. Despite these limitations, the convergent evidence from a highly suggestive clinical course, a high-probability clinical score, and strongly positive serology makes the diagnosis of HIT the most parsimonious and clinically actionable explanation for this patient's deterioration. This case powerfully illustrates the imperative to interpret unexplained heparin resistance with thrombosis as a critical pre-thrombocytopenic harbinger of HIT.

## 4 Conclusion

In summary, this case report vividly illustrates that unexplained heparin resistance accompanied by recurrent or progressive thrombosis can be a critical, yet easily overlooked, harbinger of HIT, often preceding overt thrombocytopenia. Our experience underscores the peril of responding to such resistance with further heparin dose escalation. Instead, it mandates an immediate shift in clinical reasoning: high suspicion for HIT, prompt laboratory evaluation (e.g., 4Ts score and anti-PF4/heparin antibody testing), and the expeditious initiation of a non-heparin anticoagulant, such as a direct thrombin inhibitor. Recognizing this atypical presentation is paramount to preventing life-threatening thrombotic complications and improving patient outcomes in HIT.

## Data Availability

The original contributions presented in the study are included in the article/Supplementary material, further inquiries can be directed to the corresponding author.
