# Supplementary material for: Case Report: Heparin resistance as the harbinger of heparin-induced thrombocytopenia in acute pulmonary embolism
Source: Front Med (Lausanne). 2026 Jun 18;13:1818171. doi: 10.3389/fmed.2026.1818171 (PMC13323304; doi:10.3389/fmed.2026.1818171)
Supplement: Supplementary file 1 [file Table_1.docx]

**Supplementary**

**Supplementary Table S1. Timeline of Key Clinical Events, Laboratory Parameters, and Interventions.**

| **Date (YYYY-MM-DD)** | **Heparin Tx Day** | **Key Clinical Events / Interventions** | **Platelet Count (×10⁹/L)** | **Max IV UFH Infusion Rate (U/h)** | **Key APTT Status / Values** | **Remarks / Imaging Findings** |
| --- | --- | --- | --- | --- | --- | --- |
| 2023-01-23 | 0 | **Admission.**Initial management in the Emergency Department: **Low-molecular-weight heparin (LMWH) 5000 AXa units SC**. Upon ward admission: **IV UFH therapy commenced**. | 233 (Baseline) | **Initial dose: 1000 U/h (~13.3 U/kg/h)**​ | Monitoring initiated. Target range: 1.5-2.5 × baseline. | Diagnosis: Acute PE (intermediate-high risk), DVT, RV mass. |
| 2023-01-24 | 1 | - | - | Incrementally escalated | Subtherapeutic (target not achieved) |  |
| 2023-01-25 | 2 | - | - | Incrementally escalated | Subtherapeutic (target not achieved) |  |
| 2023-01-26 | 3 | **First hemodynamic decompensation**​ (BP 75/52 mmHg). **First rescue thrombolysis**​ (rt-PA 50 mg). The UFH infusion was held during rt-PA administration. | 240 | ~1750 U/h​ (escalated prior to thrombolysis) | Subtherapeutic (target not achieved) | After rt-PA, UFH was restarted at 1500 U/h once APTT was <2× ULN, then titrated. Echocardiography: Worsened RV function, enlarged mobile cardiac mass. |
| 2023-01-27 | 4 | - | 253 | Incrementally escalated | Subtherapeutic (target not achieved) |  |
| 2023-01-28 | 5 | - | - | Incrementally escalated | Subtherapeutic (target not achieved) |  |
| 2023-01-29 | 6 | **Second hemodynamic decompensation**​ (BP 85/45 mmHg). **Second rescue thrombolysis**​ (rt-PA 50 mg). The UFH infusion was held during rt-PA administration. | 221 | 1850 U/h​ (prior to thrombolysis) | Subtherapeutic (target not achieved) | After rt-PA, UFH was restarted at 1050 U/h once APTT was <2× ULN, then re-escalated.​  Venous duplex US: DVT extension. |
| 2023-01-30 | 7 | - | - | Incrementally escalated | Subtherapeutic (target not achieved) | platelet decline begins. |
| 2023-01-31 | 8 | **Marked platelet decline, 4T score 7 (high probability), high suspicion for HIT. All heparins discontinued. IV Argatroban infusion commenced**​ (starting dose 2 μg/kg/min). | 104 | 0 (Discontinued) | Target range achieved promptly with argatroban (49.8-99.6 s) | Blood sample sent for anti-PF4/heparin IgG antibody. |
| 2023-02-01 | 9 | **Anti-PF4/heparin IgG antibody confirmed strongly positive (OD 1.92). Acute HIT diagnosed.**​ Argatroban dose titration. | 81 (Nadir) | 0 | Therapeutic range (argatroban) | Argatroban therapy effective. |
| 2023-02-02 | 10 | Argatroban infusion continued. | 85 | 0 | Therapeutic range (argatroban) | Clinically stable. |
| 2023-02-07 | 15 | **Platelet count >150 × 10⁹/L. Argatroban discontinued. Switched to oral Rivaroxaban**​ (15 mg twice daily). | 152 | 0 | N/A (Oral anticoagulant) | Successful transition to oral therapy. |
| 2023-02-13 (Pre-discharge) | 21 | Follow-up prior to discharge. | 374 | 0 | N/A | CTPA: Improvement in bilateral pulmonary emboli. Echocardiography: Normal RV function, no intracardiac mass. |
| 2023-06-06 (Follow-up) | - | Outpatient follow-up. | Within normal range | 0 | N/A | CTPA: Complete resolution of thrombus in the pulmonary trunk and main branches; residual filling defect in the right upper lobe branch. |

**Supplementary Table S2. Serial Platelet Count Monitoring During Hospitalization.**

| **Time of Sampling** | **Time (Day)** | **Platelet Count(×10⁹/L )** |
| --- | --- | --- |
| 2023/1/23 21:45 | 0 | 233 |
| 2023/1/27 5:55 | 3.34 | 240 |
| 2023/1/28 6:07 | 4.35 | 253 |
| 2023/1/30 6:15 | 6.35 | 221 |
| 2023/1/31 6:08 | 7.35 | 104 |
| 2023/2/1 6:42 | 8.38 | 82 |
| 2023/2/1 17:03 | 8.8 | 82 |
| 2023/2/2 6:50 | 9.38 | 81 |
| 2023/2/3 6:11 | 10.35 | 85 |
| 2023/2/3 13:33 | 10.66 | 84 |
| 2023/2/4 6:20 | 11.36 | 86 |
| 2023/2/4 17:44 | 11.83 | 85 |
| 2023/2/5 6:15 | 12.35 | 95 |
| 2023/2/5 17:26 | 12.82 | 93 |
| 2023/2/6 6:46 | 13.38 | 113 |
| 2023/2/6 14:43 | 13.71 | 135 |
| 2023/2/7 6:49 | 14.38 | 152 |
| 2023/2/7 13:34 | 14.66 | 164 |
| 2023/2/10 6:36 | 17.37 | 331 |
| 2023/2/13 7:00 | 20.38 | 374 |
